# Optical histopathology based on the nonlabeling analysis with multiphoton excitation imaging

**DOI:** 10.1111/pin.13498

**Published:** 2024-12-11

**Authors:** Takahiro Matsui

**Affiliations:** ^1^ Department of Pathology Graduate School of Medicine, Osaka University Osaka Japan

**Keywords:** artificial intelligence, autofluorescence, multiphoton excitation imaging, second harmonic generation, third harmonic generation

## Abstract

Histopathological diagnosis is the definitive method for the evaluation of disease status; however, some problems need to be solved, such as invasiveness, time consumption, and difficulty in three‐dimensional observation. To overcome these problems, a novel observation method, distinct from conventional histology, using tissue sections and glass slides is desirable. Fluorescence imaging of human tissues with multiphoton excitation imaging (MpEI), which was originally used for intravital imaging in biological research, is a promising method. Label‐free MpEI, which requires only near‐infrared excitation, can construct images with autofluorescent signals from fresh tissues, as well as nonlinear optical phenomena. It is possible to perform real‐time three‐dimensional imaging of human tissues without any tissue removal, fixation, or staining. This method has been reported to be useful for histopathological classification in multiple organs and tissues. Moreover, it is very compatible with quantitative image analyses, including artificial intelligence. Based on these characteristics, label‐free MpEI has sufficient potential for clinical applications such as in endoscopy and intraoperative rapid diagnosis. The clinical application of label‐free MpEI will bring changes not only to histopathology examination but also the clinical bedside and will contribute to the further development of histopathology.

**Abbreviations**
AI: artificial intelligence
MpEI: multiphoton excitation imaging
SHG: second‐harmonic generation
THG: third‐harmonic generation

## INTRODUCTION

The morphology of the cells and tissues contains invaluable information. Histopathologists recognize that various pieces of information reflecting life phenomena are condensed in the cells and tissues, although retrieving these information requires training and experience. Histopathologists may be regarded as professionals who extract and verbalize important elements related to the disease from these enormous and diverse morphological data. Since the establishment of histopathology by Dr. Rudolf Virchow et al. in the 19th century, hematoxylin and eosin‐stained specimens with thin‐sliced tissues on glass slides have been the primary tools for histological analysis. Although this modality is undoubtedly the most established method, supported by over a century of knowledge accumulation, some hurdles remain to be overcome. First, conventional histopathological examinations inevitably involve an invasive tissue removal step. Although the vast majority of procedures are performed safely without complications,^1^ it is preferable to examine them with minimal risk of tissue damage, complications, and pain. Moreover, we could not obtain information on morphological changes over time after the lesion was excised. Continuously observing the tissue in a time‐lapse manner without excising it from the living body would significantly contribute to histopathological interpretation. Second, a substantial amount of time is required from tissue removal to histological diagnosis due to the multiple steps involved in preparing glass slides from tissue samples, including fixation, dehydration, embedding, and staining.^2^ Improving the “turnaround time” problem would expedite clinical intervention and offer significant advantages.^3^ Therefore, a real‐time histopathological diagnosis is ideal. Third, conventional histology can only provide planar two‐dimensional images on glass slides, despite the excised tissue having three‐dimensional information. This limitation indicate that some morphological information is lost, which may impact diagnostic accuracy.^4^ Therefore, three‐dimensional histopathological evaluation might increase the diagnostic sensitivity. Finally, most current histopathological diagnoses depend on qualitative classification by experienced pathologists, and continuing to train high‐quality professionals is indispensable for maintaining diagnosis quality. Recently, several attempts have been made to incorporate image classification technology based on artificial intelligence (AI) for histopathological diagnosis. Image classification with quantification is crucial both scientifically and for maintaining quality. Thus, a tissue observation method that supports such a modality is also important. Developing a novel real‐time tissue observation method involving fewer and less invasive steps is desirable.

Recent advancements in basic life sciences have led to the establishment of novel observation techniques, facilitating a more vivid and detailed examination of life phenomena. Notably, fluorescence‐based observation tools, which originated from the discovery of green fluorescent proteins by Dr. Osamu Shimomura,^5^ have been particularly significant. Fluorescent proteins with various color tones, brightness, and functions are now available. These proteins function not only as markers for cells and molecules, but also as important biosensors for monitoring cellular and molecular activities, such as fluorescence‐labeled cell cycle proteins for real‐time tracking of the cell cycle,^6^ apoptosis visualization through fluorescence resonance energy transfer,^7^, ^8^ and pH‐activatable probes.^9^ These fluorescent biosensors enable the visualization of biological phenomena in live cells without pretreatment. Furthermore, intravital imaging technology has been established to observe not only the cells but also animal tissues or organs. A significant distinction exists between the importance of life phenomena observed in vitro and in vivo. Cell behaviors observed directly in vivo are considered to precisely reflect the actual state of life. Intravital imaging technology is a tool for observing living cells in real‐time in the living body and is an indispensable method for researchers in elucidating life phenomena in vivo. Multiphoton excitation imaging (MpEI) is a typical intravital imaging technique. MpEI has contributed to the elucidation of various life phenomena such as amyloid plaque formation in Alzheimer's disease,^10^ and the migration mechanism of osteoclasts in the bone.^11^ In this review, I would like to outline the characteristics of fluorescent intravital imaging and MpEI, and introduce its application to histopathology as well as its prospects.

## PRINCIPLE AND CHARACTERISTICS OF MpEI

**Figure 1:**
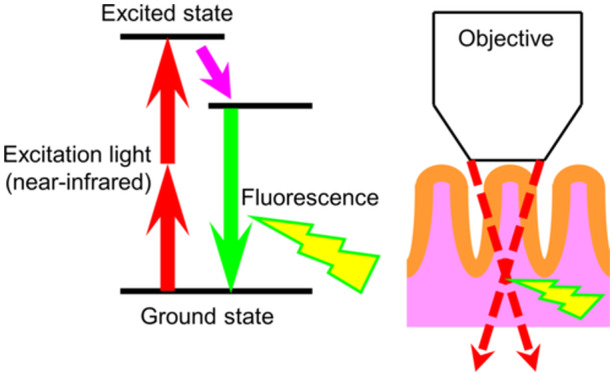
Schematic images of the principle of multiphoton excitation imaging (MpEI). Multiple near‐infrared lights are irradiated simultaneously, leading to excitation only at the focal plane where photons are densely concentrated. The employment of near‐infrared also makes it possible to observe the excitation phenomenon deep inside the tissue.

Denk et al. first reported the optical principles of the MpEI in 1990.^12^ In most fluorescence imaging techniques, it is common to label cells or molecules of interest using fluorescent proteins or dyes. In general, fluorophores are energetically stable in their ground state without light absorption. However, energetic activation by excitation light waves brings a fluorophore to its maximal energy level, called the excited state. The fluorophore then undergoes a conformational change; the electrons fall to a lower, more stable energy level, and some of the absorbed energy is released as heat. The electrons subsequently return to their ground state, releasing the remaining energy as fluorescence. The most significant feature of MpEI is that fluorescence is generated by multiple excitation photons absorbing simultaneously, instead of a single excitation photon (Figure 1). This multiple‐photon excitation rarely occurs in nature, but extreme augmentation of photon density using a femtosecond pulsed laser can increase the occurrence probability.^13^ Fluorescence by multiphoton excitation is generated only on the focal plane of the objective lens, where the excitation light is focused, thereby obtaining a high‐resolution image by the confocal effect. Moreover, near‐infrared lasers, which have excellent permeability in living tissues, are available in MpEI because the excitation wavelength may be doubled. This makes it possible to obtain tissue images at a depth of several hundred micrometers from the surface with minimal damage compared to fluorescent imaging with a single excitation light, such as confocal microscopy.^14^

In summary, MpEI has the following three major advantages: (1) high spatial resolution, (2) high tissue permeability, and (3) low tissue invasiveness. These characteristics are extremely useful for observing tissues and organs intravitally, and have made it possible to observe with sufficient resolution, in deep regions, and for a longer time, compared to conventional fluorescence microscopes. Moreover, the decisive feature of intravital imaging, including MpEI, is that we can obtain not only positional information (three‐dimensional consisting of x‐y‐z axes), but also four‐dimensional biological information, including the time axis. That is, it is possible to detect “when,” “where,” and “how” the cells work in the living body by visualizing movement, function, and differentiation in real time.^15^, ^16^, ^17^, ^18^, ^19^, ^20^, ^21^, ^22^, ^23^

## LABEL‐FREE MpEI OF HUMAN TISSUE FOR HISTOPATHOLOGY

The characteristics of MpEIs mentioned above are also advantageous when observing human tissues for histopathological evaluation. Recently, many attempts to apply fluorescence imaging to clinical histopathology have been reported.^24^, ^25^ However, one of the biggest obstacles to applying fluorescence imaging to human tissues is that it is impossible to label cells or molecules with fluorescent proteins and dyes. Fluorescent labeling by genetic modification, which is widely used in biological research, is impossible in the clinic, and the clinical administration of fluorescent reagents may be difficult, except for a few fluorescent reagents.^26^, ^27^ For clinical applications, label‐free fluorescence imaging is the most realistic and compatible method. In living cells, there are various fluorescent substances such as coenzymes reduced nicotinamide adenine dinucleotide, flavin adenine dinucleotide, and the amino acid tryptophan, emitting so‐called “autofluorescence” even without dye staining.^28^, ^29^ Therefore, label‐free imaging can be performed effectively by utilizing autofluorescence signals.

Nonlinear optics is another useful fluorescent signal for label‐free imaging. When a material is exposed to extremely strong light, it responds differently than when exposed to weak (conventional) light. The development of coherent light such as a laser has made it possible to concentrate the energy of light temporally and spatially and observe various phenomena, such as nonlinear optics, that would not occur with weak light only. The most important phenomenon among these is second‐harmonic generation (SHG), a nonlinear scattering effect that generates light of steep peak with exactly half the excitation wavelength.^30^ In intravital imaging, it is important that aligned fibrous collagen structures such as collagen type 1 generate SHG signal.^31^ In addition, other nonlinear optics such as third‐harmonic generation (THG)^32^ can also be useful signals in label‐free imaging. The detailed mechanism of THG occurrence remains unclear; however, previous reports have indicated that the existence of an interface is an important factor.^32^, ^33^ Such nonlinear optics makes it possible to visualize the alignment state and arrangement structure of molecules with high contrast.^34^ Utilizing these optical properties, label‐free fluorescence imaging is considerably effective, even when the sample is alive. Moreover, it is linked to the side benefits of omitting several steps such as tissue sampling, fixation, and staining.

There are other nonlabeled imaging methods for living tissues. For example, Raman spectroscopy utilizes the vibrational properties and distribution of components, such as proteins, lipids, and nucleic acids, to generate contrast. Although previous studies have reported the value of label‐free detection by Raman spectroscopy for guiding the surgical margin,^24^ the images are not clear, and the depth of the imaged regions is currently limited compared with MpEI. Photoacoustic microscopy, which is based on a hybrid technique of optical excitation and ultrasound detection, is another tool for nonlabeled imaging.^35^ It has been proven useful in the analysis of human samples^36^; however, advanced fluorescent imaging techniques, including MpEI, seem more advantageous for future applications in clinical medicine in terms of spatiotemporal resolution.^37^ Therefore, this review describes the results of studies focusing on the usefulness of label‐free imaging with MpEI.

### Colorectal tissue and cancer

**Figure 2:**
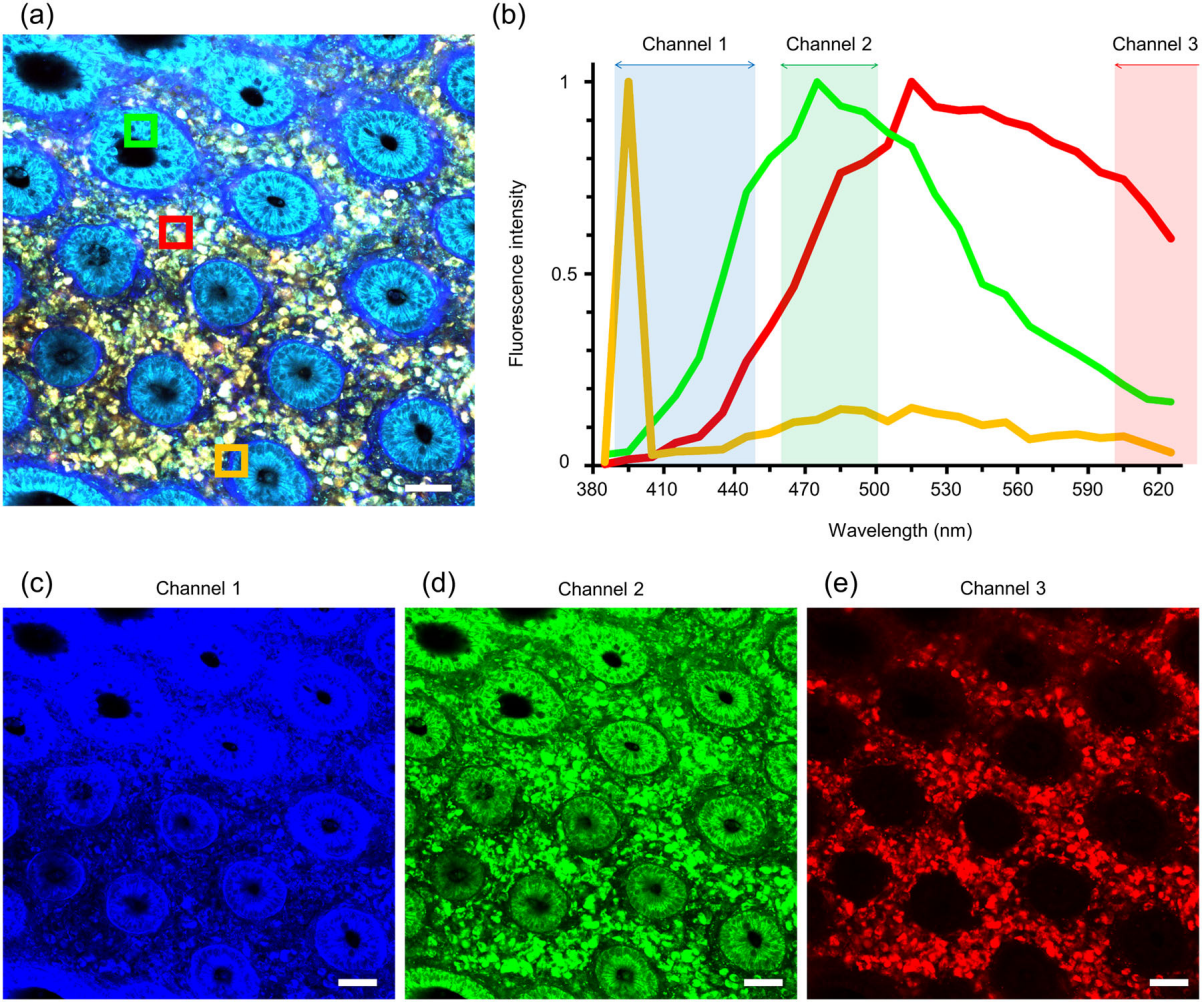
The composition of multiphoton excitation imaging (MpEI) images of human colorectal mucosa. (a and b). The MpEI image (a) and the representative fluorescence patterns of the normal colorectal mucosa (b). The MpEI image consists of mainly three areas; 1. Ductal epithelium (indicated in green square in (a) and line in (b)), 2. Blood cells in lamina propria (indicated in red), and 3. basement membrane (indicated in orange). Each component shows a different fluorescence pattern when irradiated with excitation light (780 nm), as shown in (b). (c–e). The fluorescence signals in the three wavelength ranges, indicated in (b), are respectively imaged with blue (c; 387–447 nm), green (d; 460–500 nm) and red (e; 601–657 nm). The MpEI image of (a) is a superposition of these three images. The MpEI images were taken with the imaging system consisted of an upright multiphoton microscope (A1RMP+, Nikon) driven by a laser tuned to 780 nm. Raw imaging data were processed using the NIS‐element software (Nikon). Bar: 50 μm.

**Figure 3:**
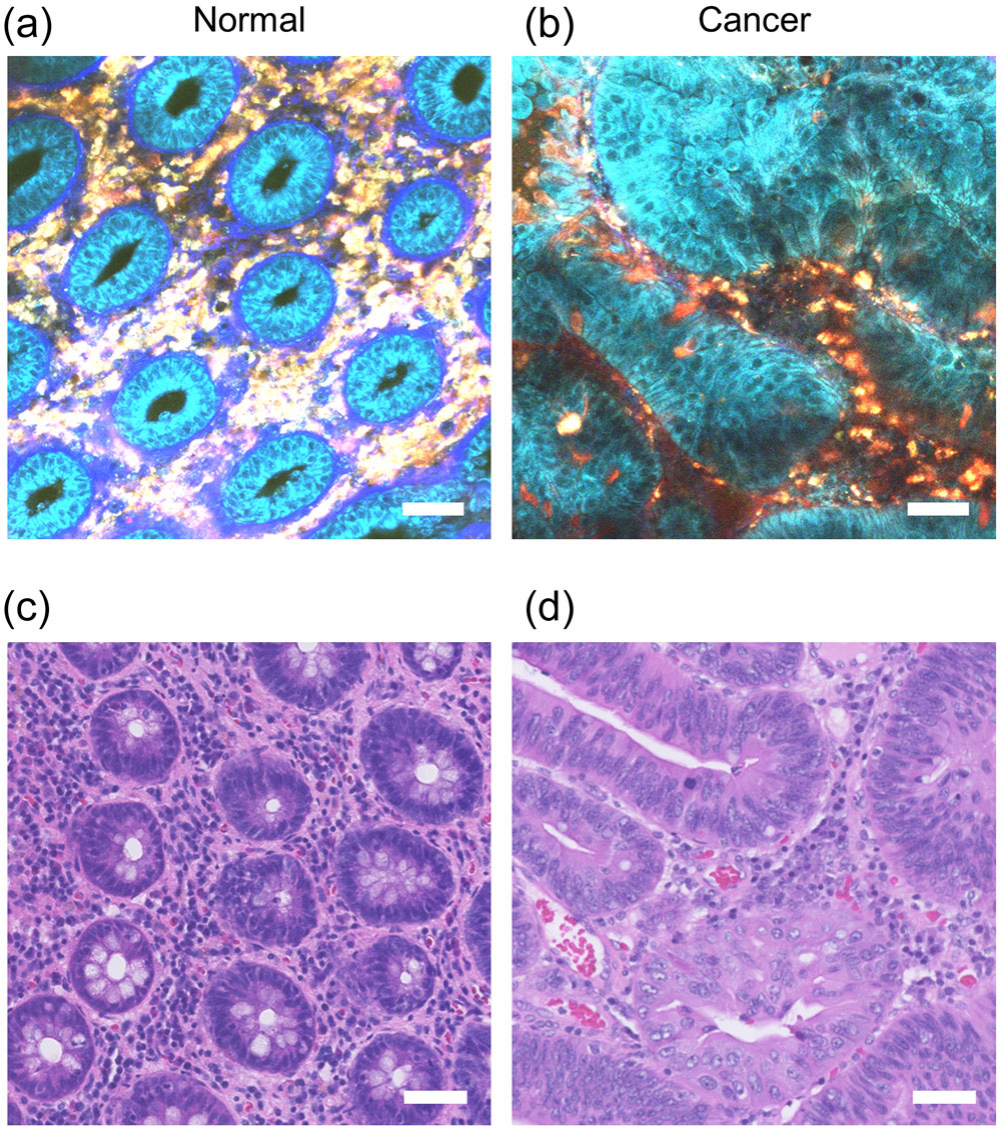
Representative images of multiphoton excitation imaging (MpEI) imaging of colorectal tissues. (a and b). Representative images of MpEI imaging of normal colon mucosa (a) and colon cancer tissue (b). The MpEI images were taken with the imaging system consisted of an upright multiphoton microscope (A1RMP+, Nikon) driven by a laser tuned to 780 nm. Raw imaging data were processed using the NIS‐element software (Nikon). (c and d). Hematoxylin and eosin‐stained images of normal colon mucosa (c; the same sample as [a]) and colon cancer tissue (d; the same sample as [d]). Bar: 50 μm. Panels are reproduced from my previous publication^30^ under the terms of the Creative Commons Attribution 4.0 International Public License (CC BY 4.0).

The lamina propria of the colorectal tissue mainly consists of columnar epithelial cells, immune cells, and collagen fibers that constitute the basement membrane. In fresh human colorectal mucosa, autofluorescence from epithelial cells peaks at approximately 460 nm with minimal detection in the region above 600 nm under an excitation wavelength of 730 nm.^30^ Spectral analyses at different excitation wavelengths indicated that sufficient emission signals could not be obtained under excitation at longer wavelengths (820 and 900 nm). These characteristics are consistent with those of autofluorescence due to the reduced pyridine nucleotides: nicotinamide adenine dinucleotide and nicotinamide adenine dinucleotide phosphate.^29^ Cellular nuclei were identified as signal‐void regions against the background of fluorescent cytoplasm. In contrast, autofluorescence from immune cells in the lamina propria peaked after 500 nm and was sufficiently detected even in the region above 600 nm under 730 nm excitation.^30^ Spectral analysis with different excitation wavelengths indicated that similar emission signals were detected under excitation at longer wavelengths (820 and 900 nm). These characteristics are consistent with those of autofluorescence due to flavin adenine dinucleotide.^29^ In addition, basement membrane beneath the crypts, which is abundant of collagen fibers, was visualized using SHG, which produces light of steep peak with exactly half the excitation wavelength.^30^ Such wavelength shifts in multiple fluorescent signals can be clearly visualized by dividing the wavelength range, imaging the fluorescence intensities in each range, and overlapping them (Figure 2). In colorectal mucosa, superimposing the three fluorescent images (blue, green, and red), which represented fluorescent signals in different wavelength ranges, facilitates identification of important histological components (e.g., ductal epithelium, immune cells, basement membrane) in different colors. Moreover, such MpEI images can describe histopathological differences. In colorectal cancer tissues, it is possible to detect nuclear enlargement of tumor cells (Figure 3). In addition, we found that the SHG signal from the basement membrane beneath the ductal structures was diminished in cancer tissue.^30^ By recognizing these morphological features, experienced pathologists can distinguish normal and cancerous tissues with high accuracy using only MpEI images.^30^

### Breast tissue and cancer

**Figure 4:**
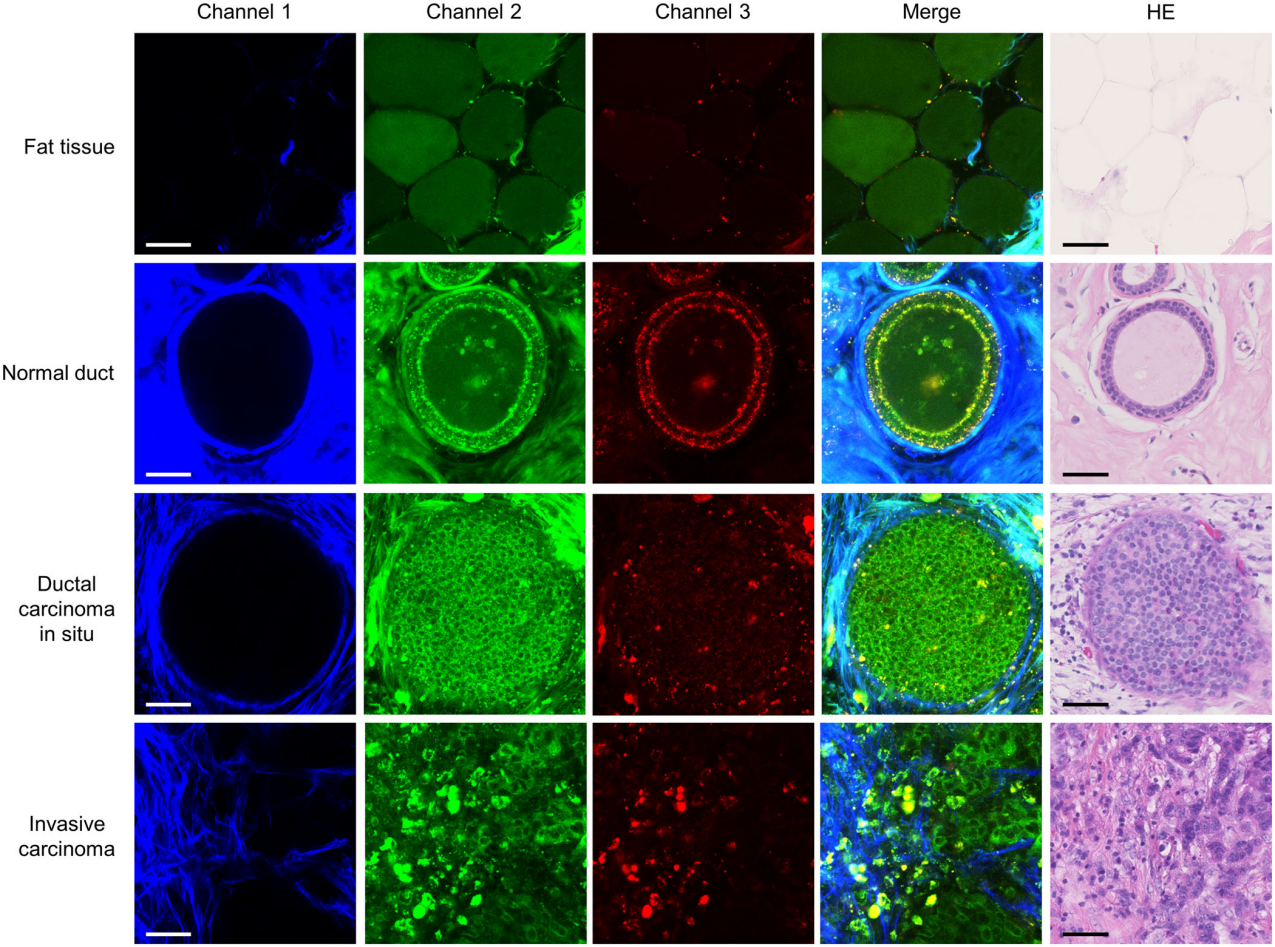
Representative images of multiphoton excitation imaging (MpEI) imaging of breast tissue with various histological types. The MpEI images were taken with the imaging system consisted of an upright multiphoton microscope (A1RMP+, Nikon) driven by a laser tuned to 780 nm. Raw imaging data were processed using the NIS‐element software (Nikon). Merged fluorescent images (fourth column) are constructed by the superposition of three fluorescent images from different channels. Hematoxylin and eosin (HE)‐stained images in the fifth column were captured after the imaging analysis from the same tissue. Bar: 50 μm. Panels are reproduced from my previous publication^38^ under the terms of the Creative Commons Attribution 4.0 International Public License (CC BY 4.0).

The same procedure used for colorectal tissue imaging can be used similarly to human breast tissue. By setting three wavelength ranges to image each fluorescence intensity, it was possible to describe various types of histopathological characteristics, such as adipose tissue, normal ducts, ductal carcinoma in situ, and invasive ductal carcinoma, after superimposing the three images (Figure 4).^38^ Interestingly, lipid droplets in adipose cells can also be recognized as fluorescence‐positive regions, unlike hematoxylin and eosin‐stained images, which exhibits only vacuolar components because label‐free imaging is performed under fresh conditions without the defatting process. In addition, a fluorescent signal at approximately 600 nm was observed around the non‐neoplastic breast ducts and lobules, showing a distribution similar to that of myoepithelial cells. In contrast, enlarged nuclei and disordered structures were observed in the neoplastic tissues, as well as colorectal carcinoma tissues.

### Uterine cervix and cervical cancer

**Figure 5:**
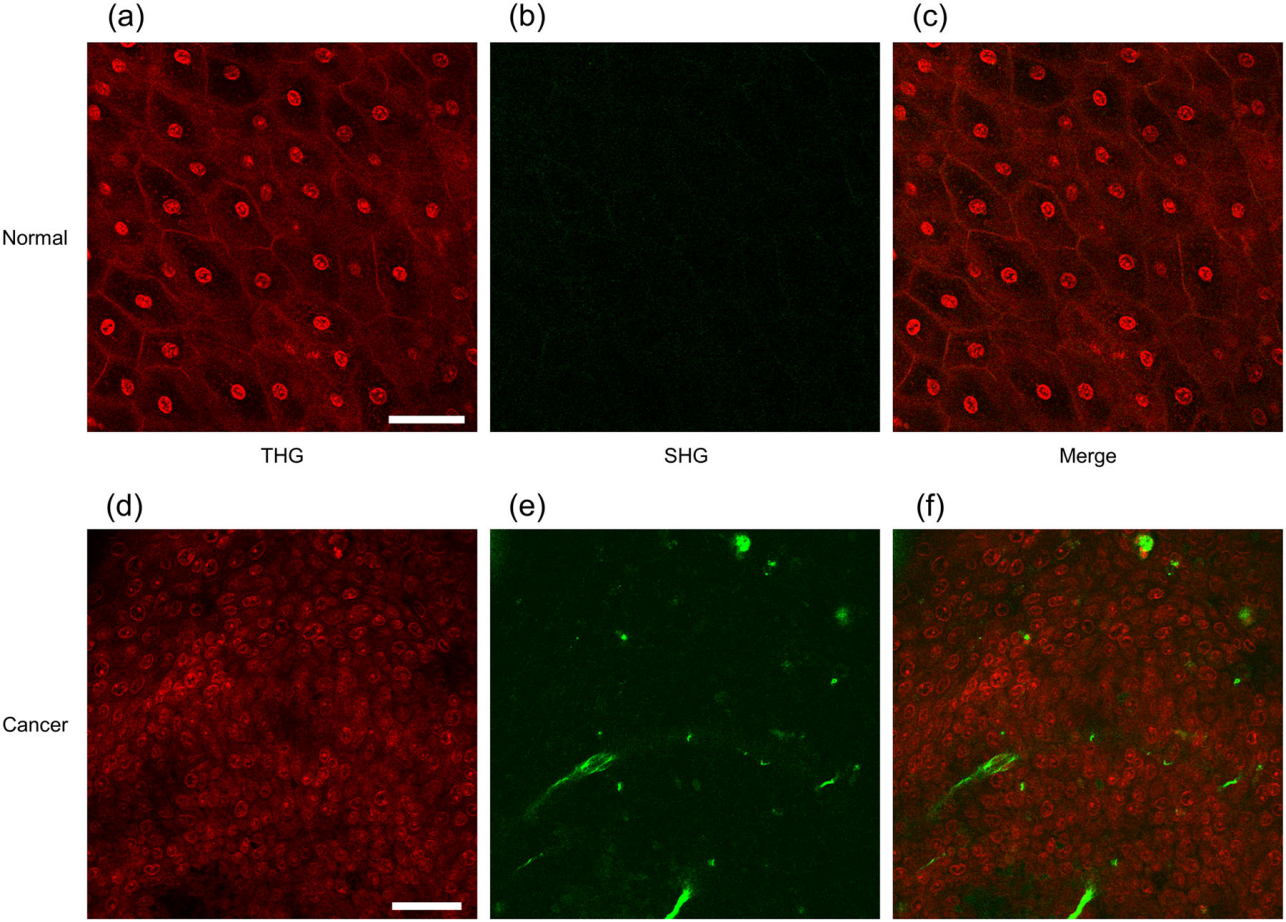
Representative images of multiphoton excitation imaging (MpEI) imaging of human normal (a–c) or malignant (d–f) cervical epithelium. The MpEI images were taken with the imaging system consisted of an upright multiphoton microscope (A1RMP+, Nikon) driven by a laser tuned to 1170 nm. Raw imaging data were processed using the NIS‐element software (Nikon). Red and green images indicate THG and SHG signals, respectively. Merged fluorescent images (third column) are constructed by the superposition of two fluorescent images. Bar: 50 μm.

An imaging method using nonlinear optics alone, without autofluorescent signals, has also been reported for the uterine cervix. As mentioned above, SHG is suitable for the visualization of fibrous collagen structures, including collagen type 1, which is also increased by cancer invasion and subsequent desmoplastic reactions. Therefore, tissue images of SHG signals are useful for estimating cancer invasion. THG is a nonlinear optical phenomenon, and although THG imaging of human cervical tissue is vague without treatment, the administration of acetic acid—widely used in gynecological clinical settings—enables the visualization of epithelial cellular nuclei with THG signal.^39^ This indicates that atypia of nuclear morphology can be detected by label‐free MpEI without tissue sampling or staining with nuclear stain reagents (Figure 5).

## MpEI AND THE AFFINITY FOR QUANTITATIVE ANALYSES OF FLUORESCENT DIGITAL IMAGES

Label‐free MpEI for human tissues enables minimally invasive, multilayered, and real‐time histopathological evaluation and offers easy access with excellent affinity for quantitative image analyses, such as AI. In conventional histopathology, tissue image data originally exists as analog images on glass slides. Therefore, image digitization processes, such as whole‐slide imaging, are necessary to perform quantitative image analysis. In contrast, in MpEI, the raw data consist of the superposition of multiple digital fluorescence images. Therefore, we can apply the quantitative analysis modality immediately after the MpEI procedure. The accessibility of such digital tools for MpEI is promising enough to open the possibility of quantitative evaluation without pathologist intervention.

**Figure 6:**
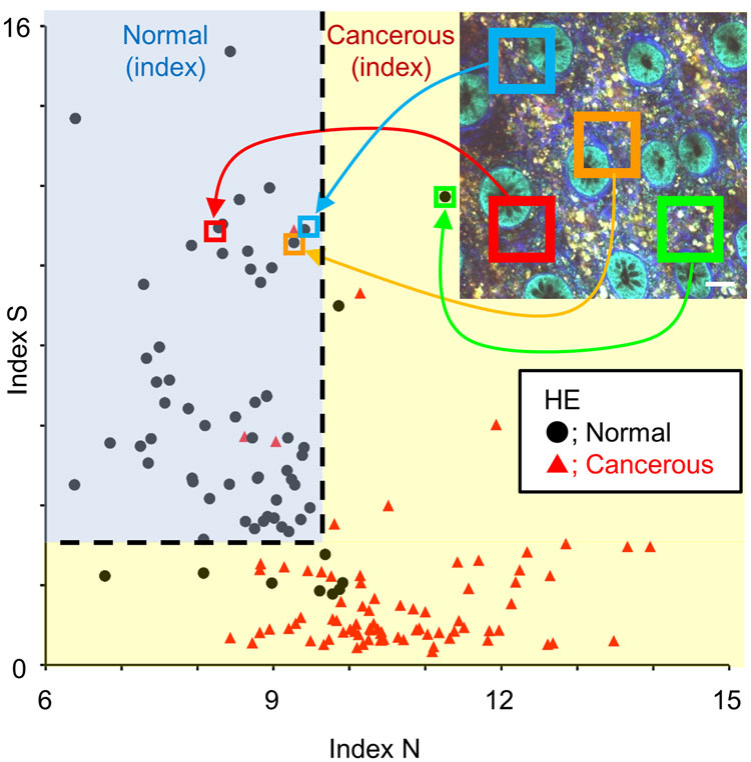
Representative scheme of the scatter plot from the classification analysis using two feature values. Indices N and S represent the mean nuclear diameter in the square region of interest (shown in the MpEI image) and the intensity of fibrous SHG signal in the square region of interest, respectively. Final classifications of normal (black dots) or cancer tissue (red triangles) are made using hematoxylin and eosin (HE)‐stained sections from the same specimens imaged by MpEI. Areas that showed an index N ≤ 9.5 and index S > 3.1 (upper left area of the dashed line) are deemed normal in the classification analysis. Bar: 50 μm. Panels are reproduced from my previous publication^30^ under the terms of the Creative Commons Attribution 4.0 International Public License (CC BY 4.0).

Several methods are available for quantitatively classifying digital image data. First, such quantitative image analysis includes the evaluation utilizing feature values extracted from the histological images. It is possible to classify images quantitatively, even if we cannot obtain a large dataset, when we can numerically distinguish the morphological differences between cancerous and non‐cancerous images as feature values. For example, in images of colorectal mucosal tissues, the cellular nuclei of columnar epithelial and cancer cells were identified as signal‐void regions against the background of fluorescent cytoplasm. Therefore, we can obtain the nuclear size of epithelial or tumor cells, which is an important feature value, from MpEI fluorescent images. In fact, the nuclear size of colorectal cancer cells was significantly larger than that of epithelial cells in normal tissues in MpEI images.^30^ The intensity of the SHG signal from the basement membrane is another important value calculated from the fluorescence intensity. SHG signals from the basement membrane beneath the ductal structures are obvious in the normal mucosa, whereas the equivalent signal is diminished in cancer tissues. As the SHG signal is the light of a steep peak with exactly half the excitation wavelength, we could quantitatively evaluate the visual intensity of the SHG signal by measuring the fluorescence intensity over narrow wavelength ranges.^30^ By combining these two feature values, MpEI fluorescence images of colorectal tissues can be classified into non‐cancerous or cancerous tissues without pathologist intervention (Figure 6).^30^

If we pursue manual‐free analysis, it is possible to employ AI to extract regions of interest from fluorescence images. For example, in the MpEI of the uterine cervix, the shapes of the epithelial and cancer cell nuclei can be visualized through THG imaging. After utilizing a deep learning system that enables automatic detection of cellular nuclei, THG images of cervical tissue can express the nuclear atypia of cervical tumor cells with several feature values, including maximum area, nuclear density, and circularity. In situations where multiple feature values can be set, precise classification can be performed using a machine‐learning model.^39^ Using analytical procedures with either SHG (indicating fibrosis due to tumor invasion) or THG (indicating nuclear atypia) images alone, normal and cancerous tissues were classified quantitatively, with an area under the curve of >0.92. Moreover, combinatorial analysis using THG and SHG signals is useful for the accurate classification of normal cervical tissue, cervical intraepithelial neoplasia, and invasive cervical carcinoma.^39^ In these classification methods that use feature values, it is important to explain the classification criteria.

It is not beyond the capabilities of a deep learning system to complete everything up to image classification. MpEI fluorescent images also have good affinity for classification with deep learning, which is free from feature value settings. The usefulness of classifying and analyzing image data using deep learning is well‐established in medical image analysis.^40^ In conventional histological images, such as hematoxylin and eosin‐stained images, the application of deep learning has progressed significantly. Multiple reports have suggested that deep learning analysis excels in image recognition or identification, and that algorithms with deep learning can accurately analyze or classify histological images.^41^ These properties are also applicable to the classification of fluorescence images using MpEI. In the images of breast tissue using autofluorescence signals, deep learning analysis can detect cancer cells in a small range of <100 µm square with sufficient accuracy.^38^ To construct a deep‐learning classification system, it is necessary to prepare a large amount of image data. However, in a system that detects a small number of tumor cells hidden in a small area, the analyzed image size was, in turn, small. Therefore, it may be easy to overcome the problem of image data volume because we can use large images and divide them into several images of appropriate size. In addition, the development of AI systems that can efficiently classify image data using small amounts of labeled data has also been reported recently,^42^ which may make it easier to construct efficient AI classification systems. Another issue in image classification by deep learning algorithm is the “explainability” of algorithm results. Deep learning, which is widely used in image classification AI, learns from a large amount of data using complex networks with multiple layers. Therefore, it is practically impossible for humans to understand the learning process. As a result, the basis for the AI classification decision cannot be explained, although the classification results are available. As reports of outstanding image classification using AI have increased, some studies have attempted to uncover the fundamentals of AI image classification with high accuracy. For example, methods such as gradient‐weighted class activation mapping (Grad‐CAM), which analyzes deep learning models by focusing on learning weights, have recently been used to address this issue.^43^ Alternatively, another attempt has been made to get closer to the background of the classification process by having humans reanalyze the AI classification results.^44^ However, this method remains in the category of speculation, and in principle, it is impossible to extract and understand the classification criteria. In terms of scientific analysis, quantitative and reproducible classification procedures are not considered problematic. However, in the clinical medical field, the rationale for histopathological diagnosis, as well as who should be responsible for it, becomes problematic. Nonetheless, utilizing the deep learning architecture for histopathological assessment often has the advantage of high image recognition ability, outweighing its disadvantages. Therefore, it is important to determine the extent to which AI analysis in the field of clinical medicine can be tolerated and used in clinical applications. I believe that it should be approached flexibly and continuously, depending on time, medical needs, and social conditions.

## PROMISING CLINICAL APPLICATIONS OF LABEL‐FREE MpEI

MpEI is a useful optical technology with potential for application in clinical histopathology. The MpEI has two major advantages in clinical applications: minimal invasiveness and real‐time analysis. The ability to obtain histological information in real time without tissue sampling or biopsy is sufficiently attractive for various clinical situations in many clinical departments. For example, in gastroenterology, endoscopists can obtain real‐time histological information at the bedside if an endoscope with MpEI technology is developed. Such technology would be useful in determining the resection range of early cancer lesions in endoscopic treatments such as endoscopic mucosal resection and endoscopic submucosal dissection. Therefore, endoscopy is one of the most useful options for clinical application. Another candidate for the application of MpEI is rapid intraoperative diagnosis. Most rapid intraoperative diagnoses are performed to determine the resection range during cancer surgery. It would be advantageous for surgeons to obtain such information in real‐time in the operating room. In particular, it is important to determine in reduction surgery whether the tumor cells are exposed at the resection margin. In the current rapid intraoperative diagnosis using frozen specimens, the observation range is limited because multiple procedures and considerable time are required for specimen preparation. In contrast, resection margin evaluation using MpEI can be performed in real‐time. Furthermore, in principle, it is possible to observe almost all the resection margins immediately because no tissue sampling or specimen preparation is required. Furthermore, MpEI allows observation of the “true” surgical margin on the patient's body, where it is impossible to observe in conventional frozen section.

For every application described above, many clinical benefits arise from the properties of MpEIs. First, it will help doctors promptly make decisions regarding clinical interventions using real‐time imaging. We can not only obtain the examination results of as many digital images as possible, but also access the quantitative image analysis system, including AI. In addition, the ability to observe at different focal planes by “optical slice” will provide higher dimensional information. More importantly, label‐free MpEI can coexist with conventional histopathological examinations. Although MpEI can be performed before conventional examinations (biopsy or frozen section analysis), it does not interfere with subsequent examinations. Instead, it can provide useful information before these examinations. Therefore, it may be useful as a preliminary attempt in conventional examinations. When the results of the MpEI analysis are ambiguous or deviate from clinical findings, conventional examinations can be immediately added to obtain further information.

Generally, the utility of three‐dimensional analysis relies on the analyzable depth, which varies depending on the tissues for observation. In a previous report regarding colorectal mucosa, for example, we can only evaluate the lamina propria mucosae of the colorectum (about the depth of <120 μm) using MpEI, and currently difficult to obtain clear images at greater depths.^30^ However, images from deeper regions, such as around the muscularis mucosae and submucosa, contain considerable information, such as the progression status of the malignant tumor, invasion depth, and presence/absence of vascular invasion.^45^ Moreover, some malignant tumors are difficult to detect through analysis of the superficial mucosa and often infiltrate beneath the mucosal layer, such as scirrhous gastric carcinoma.^46^ Therefore, it is necessary to examine the acquisition of images from deeper areas to develop an MpEI imaging technique as a diagnostic method for gastroenterological lesions. Conversely, imaging with nonlinear optics is possible at a depth of >500 μm in the epithelium of uterine cervix.^39^ In addition, in cervical cancer, when a tumor develops, it first grows within the epithelium and subsequently invades the subepithelial region after it has grown in almost all the epithelial layers.^47^ This indicates that MpEI has the potential to detect early cervical cancer, even within the currently reported observation range. As mentioned previously, the required optical technology varies depending on the organ, lesion, and target diseases. It is desirable to understand the characteristics of MpEIs and apply them clinically to maximize their advantages.

Single‐plane imaging with MpEI is typically completed within a few seconds. However, the higher the resolution, the longer the acquisition time required, of course. Furthermore, in three‐dimensional analysis, the imaging time varies depending on how fine the imaging width is. In other words, there exists a payoff between the amount of image information and the time required in the MpEI imaging. When considering clinical applications of MpEI, we need to take into account it to develop medical equipment.

## SUMMARY AND PERSPECTIVES

MpEI is a useful histopathological method that can cause drastic changes in histopathological diagnostic methods. This has the potential to overcome some underlying problems associated with conventional histopathology. The full‐scale clinical application of this method can potentially bring drastic changes to the clinical bedside, complement the problems of conventional examination, and contribute to the further development of histopathology.

## AUTHOR CONTRIBUTIONS

Takahiro Matsui wrote and revised the manuscript. The author gave final approval for publication.

## CONFLICT OF INTEREST STATEMENT

None declared.
